# GPR40 activation initiates store-operated Ca^2+^ entry and potentiates insulin secretion via the IP3R1/STIM1/Orai1 pathway in pancreatic β-cells

**DOI:** 10.1038/s41598-019-52048-1

**Published:** 2019-10-29

**Authors:** Ryota Usui, Daisuke Yabe, Muhammad Fauzi, Hisanori Goto, Ainur Botagarova, Shinsuke Tokumoto, Hisato Tatsuoka, Yumiko Tahara, Shizuka Kobayashi, Toshiya Manabe, Yoshihiro Baba, Tomohiro Kurosaki, Pedro Luis Herrera, Masahito Ogura, Kazuaki Nagashima, Nobuya Inagaki

**Affiliations:** 1 Department of Diabetes, Endocrinology and Nutrition, Kyoto University Graduate School of Medicine, Kyoto, Japan; 2 Department of Diabetes and Endocrinology, Gifu University Graduate School of Medicine, Gifu, Japan; 3 Yutaka Seino Distinguished Center for Diabetes Research, Kansai Electric Power Medical Research Institute, Kobe, Japan; 4 Division of Molecular and Metabolic Medicine, Department of Physiology and Cell Biology, Kobe University Graduate School of Medicine, Kobe, Japan; 5 Division of Neuronal Network, Department of Basic Medical Sciences, Institute of Medical Science, University of Tokyo, Tokyo, Japan; 6 Division of Immunology and Genome Biology, Department of Molecular and Structural Biology, Medical Institute of Bioregulation, Kyushu University, Fukuoka, Japan; 7 Laboratory of Lymphocyte Differentiation, WPI Immunology Frontier Research Center, Osaka University, Osaka, Japan; 8 Department of Genetic Medicine and Development, University of Geneva Medical School, Geneva, Switzerland

**Keywords:** Diabetes, Metabolic diseases

## Abstract

The long-chain fatty acid receptor GPR40 plays an important role in potentiation of glucose-induced insulin secretion (GIIS) from pancreatic β-cells. Previous studies demonstrated that GPR40 activation enhances Ca^2+^ release from the endoplasmic reticulum (ER) by activating inositol 1,4,5-triphosphate (IP3) receptors. However, it remains unknown how ER Ca^2+^ release via the IP3 receptor is linked to GIIS potentiation. Recently, stromal interaction molecule (STIM) 1 was identified as a key regulator of store-operated Ca^2+^ entry (SOCE), but little is known about its contribution in GPR40 signaling. We show that GPR40-mediated potentiation of GIIS is abolished by knockdown of IP3 receptor 1 (IP3R1), STIM1 or Ca^2+^-channel Orai1 in insulin-secreting MIN6 cells. STIM1 and Orai1 knockdown significantly impaired SOCE and the increase of intracellular Ca^2+^ by the GPR40 agonist, fasiglifam. Furthermore, β-cell-specific STIM1 knockout mice showed impaired fasiglifam-mediated GIIS potentiation not only in isolated islets but also *in vivo*. These results indicate that the IP3R1/STIM1/Orai1 pathway plays an important role in GPR40-mediated SOCE initiation and GIIS potentiation in pancreatic β-cells.

## Introduction

GPR40/FFAR1 signaling has been an attractive therapeutic target for the management of type 2 diabetes (T2D), as GPR40 is highly expressed in pancreatic β-cells^1^ and can potentiate glucose-induced insulin secretion (GIIS) when activated by long-chain free fatty acids such as palmitate and oleic acid, with limited hypoglycemia risk^2^. GPR40 overexpression in pancreatic β-cells augments GIIS and improves glucose tolerance^3^, and GPR40 deficiency in pancreatic β-cells impairs insulin secretion^4^. While the mechanisms underlying long-chain fatty acid enhancement of GIIS through GPR40 still remain largely unknown, an orally available GPR40 agonist, fasiglifam (fas), has been developed that potentiates GIIS and lowers blood glucose levels in diabetic animal models^5,6^. Clinical trials have demonstrated that fas significantly improves glycemic control in T2D individuals, with minimal risk of hypoglycemia^7^, but their development was terminated because of liver toxicity in phase 3 trial^8^. Despite this serious problem, several novel GPR40 agonists have been developed recently for use in animal models that have potential as new treatment modalities^9,10^.

Previous reports have recognized the involvement of various pathways in GPR40-mediated GIIS potentiation. For example, when a ligand binds to GPR40, the Gα subunit is activated by GDP/GTP exchange and separates from the trimeric G-protein, binds to phospholipase C (PLC), and thereby increases intracellular inositol triphosphate (IP3) and diacylglycerol (DAG) by hydrolysis of phosphatidylinositol 4,5-bisphosphate (PIP2)^4,11^. Ferdaoussi *et al*. reported that DAG activates protein kinase D (PKD)1 to promote actin depolymerization, which subsequently increases insulin secretion^12^. Sakuma *et al*. reported that IP3 supplement promotes the release of Ca^2+^ from the endoplasmic reticulum (ER) and potentiates GIIS in MIN6 cells^13^. However, the mechanism by which ER Ca^2+^ release leads to GIIS potentiation remains unknown.

Store-operated Ca^2+^ entry (SOCE) is initiated by depletion of Ca^2+^ from the ER, and induces extracellular Ca^2+^ influx to the cytosol to maintain intracellular Ca^2+^ homeostasis in various cell types^14,15^. SOCE is known to be regulated by stromal interaction molecule (STIM) 1, which is a membrane protein embedded in the ER^16,17^. STIM1 senses the Ca^2+^ concentration in the ER ([Ca^2+^]_ER_) by a Ca^2+^-sensing domain that projects into the lumen of the ER, and activates the Ca^2+^-channel Orai1, which is expressed on the plasma membrane, through its CRAC activation domain in the cytosol, thereby allowing robust influx of extracellular Ca^2+^^18,19^. Previous studies showed that STIM1 deficiency reduced NFAT activation and IL-10 production in B lymphocytes^20^, reduced IgE-mediated anaphylactic response in mast cells^21^, produced skeletal muscle delay in skeletal muscle^22^, and promoted insulin resistance under high-fat diet in liver^23^.

Previous studies demonstrated that STIM1 is expressed in MIN6 cells and mouse pancreatic β-cells, and that glucose or cAMP affects STIM1 translocation in β-cells^24,25^. It also was reported that SOCE regulates GIIS, using INS-1 cells^26^, but there are no reports regarding the contribution of SOCE in GPR40 signaling. Moreover, the physiological effects of STIM1 in β-cells on glucose metabolism using STIM1-deficient mice have not been studied.

As GPR40 activation results in the release of ER Ca^2+^^13^, it is not unlikely that SOCE mediates GIIS potentiation by GPR40 activation. The current study aims to establish the mechanism by which the GPR40 signal potentiates GIIS and to clarify the role of SOCE in GPR40 signaling using siRNA transfected MIN6 cells and β-cell-specific STIM1 knockout mice.

## Results

**Figure 1:**
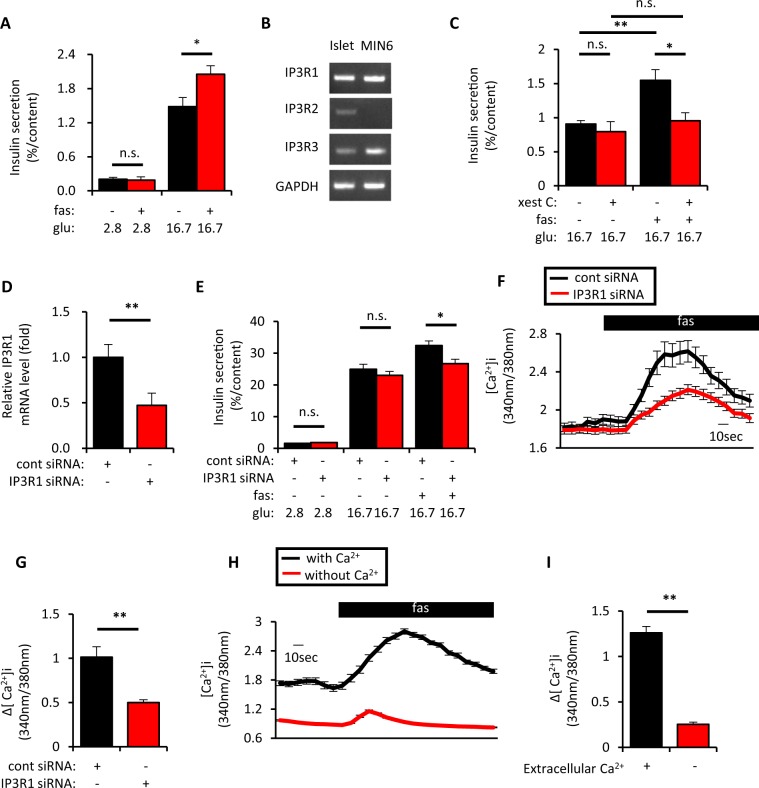
Inhibition of inositol triphosphate receptor (IP3R) 1 suppressed fasiglifam-induced intracellular Ca^2+^ elevation and potentiation of insulin secretion. (**A**) Ten isolated islets of C57BL/6 mice were collected in a tube and incubated at 2.8 mM or 16.7 mM glucose (glu) with or without 10 μM fasiglifam (fas) for 60 min at 37 °C to evaluate insulin secretion (n = 6–7 per group). (**B**) Total RNA was extracted from isolated islets of C57BL/6 mice and MIN6 cells and subjected to cDNA synthesis followed by PCR to detect mRNA expression of IP3R1, IP3R2, IP3R3 and glyceraldehyde-3-phosphate dehydrogenase (GAPDH). The uncropped image of the gel is shown in Supplemental Fig. S7. (**C**) Isolated islets were incubated as in (**A**) at 16.7 mM glu with or without 10 μM fas and 2 μM IP3 receptor (IP3R) inhibitor xestospongin C (xest C) for 60 min at 37 °C to evaluate insulin secretion (n = 5–6 per group). (**D**) MIN6 cells were set up on day 0 at 2 × 10^5^ cells per 24 well plate and transfected with 50pmol of control (cont) siRNA or IP3R1 siRNA. On day 2, total RNA was extracted from each of the transfected MIN6 cells and subjected to cDNA synthesis followed by real-time PCR to evaluate IP3R1 mRNA expression (n = 4 per group). (**E**) MIN6 cells were set up and transfected on day 0 as in (**D**). On day 2, the transfected MIN6 cells were incubated at 2.8 mM or 16.7 mM glu with or without 10 μM fas for 60 min at 37 °C to evaluate insulin secretion (n = 6 per group). (**F**) MIN6 cells were set up on day 0 at 4 × 10^5^ cells per 35 mm dish and transfected with 100pmol of cont siRNA or IP3R1 siRNA. On day 2, the transfected MIN6 cells were recorded for intracellular Ca^2+^ concentrations ([Ca^2+^]_i_) evaluated by fura-2 fluorescence ratio in response to various stimuli (−1–0 min, 11.1 mM glu; 0–3 min, 11.1 mM glu/10 μM fas). Traces show average responses of multiple transfected MIN6 cells (cont siRNA, n = 40; and IP3R1 siRNA, n = 39). (**G**) Increment of fura-2 ratio (Δ[Ca^2+^]_i_) in response to 10 μM fas in conditions as in (**F**) [(Max. value during 0–3 min) − (Average value during −1–0 min)]. (**H**) MIN6 cells were recorded for intracellular Ca^2+^ concentrations ([Ca^2+^]_i_) by fura-2 fluorescence ratio in response to fas in the absence or presence of extracellular Ca^2+^ (−1–0 min, 11.1 mM glu/0 or 2 mM Ca^2+^; 0–3 min, 11.1 mM glu/0 or 2 mM Ca^2+^/10 μM fas). Traces show average responses of multiple MIN6 cells (0 mM Ca^2+^, n = 63; and 2 mM Ca^2+^, n = 59). (**I**) Increment of fura-2 ratio (Δ[Ca^2+^]_i_) in response to 10 μM fas in (**H**) [(Max. value during 0–3 min) − (Average value during −1–0 min)]. Data are expressed as mean ± SEM in (**A**,**C**–**E**,**G**,**I**). * and ** denote p < 0.05 and p < 0.01, respectively, by the Mann-Whitney U-test.

It was previously demonstrated that GPR40 activation potentiates GIIS glucose-dependently^2^. While long-chain fatty acids such as palmitate and oleate are known to activate GPR40, fasiglifam (fas) was used in the present study to assess the GPR40 signal, as it is a widely used GPR40 agonist in previous studies. Consistent with the previous study^6^, fas potentiated insulin secretion at 16.7 mM glucose, but not at 2.8 mM glucose, in isolated mouse islets (Fig. 1A). Since GPR40 signaling is known to activate PLC and to produce IP3^5^, IP3 receptor (IP3R) involvement in fas-mediated GIIS potentiation was investigated. It was previously demonstrated in various cell types that IP3Rs activation facilitated Ca^2+^ release from the ER^27,28^, and that IP3-induced ER Ca^2+^ release is augmented when the glucose level is elevated in β-cells^29^, suggesting that they may be attractive targets to clarify the requirement of glucose in fas-mediated GIIS potentiation. Because IP3 receptor (IP3R) 1 and IP3R3 are expressed in mouse islets and MIN6 cells (Figs 1B and S1A) and IP3R1 is known to be activated by IP3 and ATP^27,30^, involvement of IP3Rs in fas-mediated GIIS potentiation was tested using the IP3R inhibitor xestospongin C. Xestospongin C significantly inhibited fas-mediated GIIS potentiation in mouse isolated islets (Fig. 1C), although it had little effect on GIIS. Knockdown experiments were also conducted in mouse insulin-secreting MIN6 cells. IP3R1 knockdown (KD) cells showed approximately 50% reduction of IP3R1 mRNA and 40% reduction of IP3R1 protein (Figs 1D, S1B and S1C). IP3R1KD significantly reduced fas-mediated GIIS potentiation and fas-mediated [Ca^2+^]_i_ increase in MIN6 cells (Fig. 1E–G).

Previous studies demonstrated that IP3R activation leads to Ca^2+^ release from the ER^27,29^. It was demonstrated in β-cells that a decline in [Ca^2+^]_ER_ by activation of IP3R allows translocation of the ER-resident Ca^2+^ sensor protein STIM1, and initiates SOCE through the Ca^2+^ channel Orai1^24,25^. Fas-mediated [Ca^2+^]_i_ change was significantly decreased in the absence of extracellular Ca^2+^ (Fig. 1H,I), which suggests that extracellular Ca^2+^ influx including SOCE contributes to the effect of fas.

**Figure 2:**
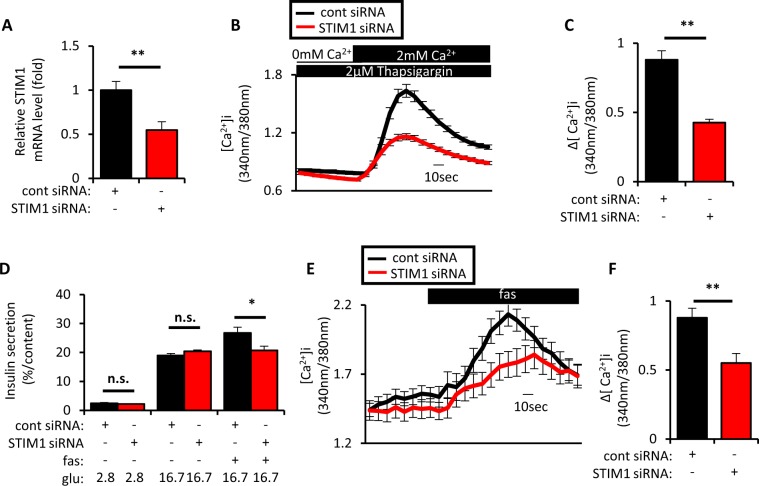
Inhibition of stromal interaction molecule 1 (STIM1) suppressed SOCE and fasiglifam-induced intracellular Ca^2+^ elevation and potentiation of insulin secretion. (**A**) MIN6 cells were set up on day 0 at 2 × 10^5^ cells per 24 well plate and transfected with 50pmol of control (cont) siRNA or STIM1 siRNA. On day 2, total RNA was extracted from each of the transfected MIN6 cells and subjected to cDNA synthesis followed by real-time PCR to evaluate STIM1 mRNA expression (n = 4 per group). (**B**) MIN6 cells were set up on day 0 at 4 × 10^5^ cells per 35 mm dish and transfected with 100pmol of cont siRNA or STIM1 siRNA. On day 2, the transfected MIN6 cells were recorded for intracellular Ca^2+^ concentrations ([Ca^2+^]_i_) to evaluate SOCE by fura-2 fluorescence ratio in response to extracellular Ca^2+^ supplementation (−1–0 min, 0 mM Ca^2+^/2 μM thapsigargin; 0–3 min, 2 mM Ca^2+^/2 μM thapsigargin). Traces show average responses of multiple transfected MIN6 cells (cont siRNA, n = 32; and STIM1 siRNA, n = 37). (**C**) Increment of fura-2 ratio (Δ[Ca^2+^]_i_) in response to extracellular Ca^2+^ supplementation in conditions as in (**B**) [(Max. value during 0–3 min) − (Average value during −1–0 min)]. (**D**) MIN6 cells were set up and transfected on day 0 as in (**A**). On day 2, the transfected MIN6 cells were incubated at 2.8 mM or 16.7 mM glu with or without 10 μM fas for 60 min at 37 °C to evaluate insulin secretion (n = 4–5 per group). (**E**) MIN6 cells were set up as in (**B**). On day 2, the transfected MIN6 cells were recorded for intracellular Ca^2+^ concentrations ([Ca^2+^]_i_) evaluated by fura-2 fluorescence ratio in response to various stimuli (−1–0 min, 11.1 mM glu; 0–3 min, 11.1 mM glu/10 μM fas). Traces show average responses of multiple transfected MIN6 cells (cont siRNA, n = 48; and STIM1 siRNA, n = 25). (**F**) Increment of fura-2 ratio (Δ[Ca^2+^]_i_) in response to 10 μM fas in conditions as in (**E**) [(Max. value during 0–3 min) − (Average value during −1–0 min)]. Data are expressed as mean ± SEM. * and ** denote p < 0.05 and p < 0.01, respectively, by the Mann-Whitney U-test.

To test involvement of STIM1 in fas-mediated GIIS potentiation, KD experiments were conducted in MIN6 cells. Approximately 50% KD of STIM1 mRNA significantly reduced SOCE (Fig. 2A–C) and fas-mediated GIIS potentiation (Fig. 2D). STIM1 KD also significantly suppressed fas-mediated [Ca^2+^]_i_ increase (Fig. 2E,F).

**Figure 3:**
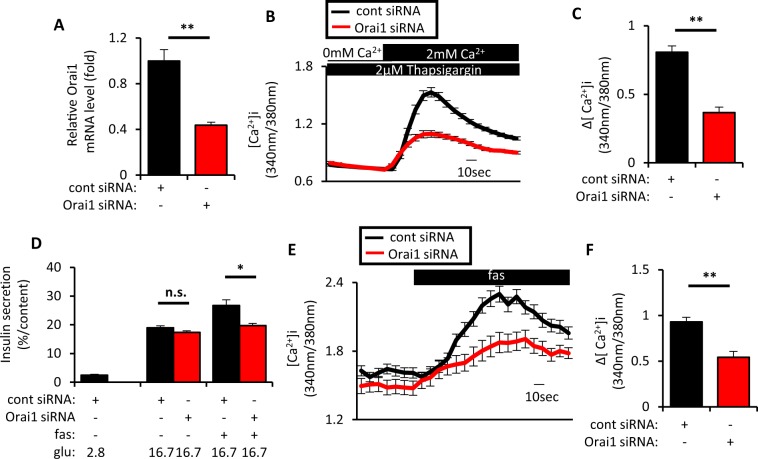
Inhibition of Orai1 suppressed SOCE and fasiglifam-induced intracellular Ca^2+^ elevation and potentiation of insulin secretion. (**A**) MIN6 cells were set up on day 0 at 2 × 10^5^ cells per 24 well plate and transfected with 50pmol of control (cont) siRNA and Orai1 siRNA. On day 2, total RNA was extracted from each of the transfected MIN6 cells and subjected to cDNA synthesis followed by real-time PCR to evaluate Orai1 mRNA expression (n = 4 per group). (**B**) MIN6 cells were set up on day 0 at 4 × 10^5^ cells per 35 mm dish and transfected with 100pmol of cont siRNA or Orai1 siRNA. On day 2, the transfected MIN6 cells were recorded for intracellular Ca^2+^ concentrations ([Ca^2+^]_i_) to evaluate SOCE by fura-2 fluorescence ratio in response to extracellular Ca^2+^ supplementation (−1–0 min, 0 mM Ca^2+^/2 μM thapsigargin; 0–3 min, 2 mM Ca^2+^/2 μM thapsigargin). Traces show average responses of multiple transfected MIN6 cells (cont siRNA, n = 56; and Orai1 siRNA, n = 48). (**C**) Increment of fura-2 ratio (Δ[Ca^2+^]_i_) in response to extracellular Ca^2+^ supplementation in conditions as in (**B**) [(Max. value during 0–3 min) − (Average value during −1–0 min)]. (**D**) MIN6 cells were set up and transfected on day 0 as in (**A**). On day 2, MIN6 cells were incubated at 2.8 mM or 16.7 mM glucose (glu) with or without 10 μM fasiglifam (fas) for 60 min at 37 °C to evaluate insulin secretion (n = 4–5 per group). (**E**) MIN6 cells were set up as in (**B**). On day 2, the transfected MIN6 cells were recorded for intracellular Ca^2+^ concentrations ([Ca^2+^]_i_) evaluated by fura-2 fluorescence ratio in response to various stimuli (−1–0 min, 11.1 mM glu; 0–3 min, 11.1 mM glu/10 μM fas). Traces show average responses of multiple transfected MIN6 cells (cont siRNA, n = 38; and Orai1 siRNA, n = 34). (**F**) Increment of fura-2 ratio (Δ[Ca^2+^]_i_) in response to 10 μM fas in (**E**) [(Max. value during 0–3 min) − (Average value during −1–0 min)]. Data are expressed as mean ± SEM. * and ** denote p < 0.05 and p < 0.01, respectively, by the Mann-Whitney U-test.

Involvement of Orai1 in fas-mediated GIIS potentiation was also investigated in downstream STIM1 signaling. As expected, approximately 60% KD of Orai1 mRNA significantly reduced SOCE (Fig. 3A–C) and fas-mediated GIIS potentiation (Fig. 3D). Orai1 KD also significantly suppressed the fas-mediated [Ca^2+^]_i_ increase in MIN6 cells (Fig. 3E,F). These results together suggest that the IP3R1/STIM1/Orai1 pathway plays a critical role in fas-mediated GIIS potentiation via activation of SOCE. Consistently, IP3R1, STIM1 and Orai1 KD also impaired GIIS potentiation when palmitate, instead of fas, was used to activate GPR40 signaling (Fig. S2A–C). These results indicate that the IP3R1/STIM1/Orai1 pathway is indispensable for GPR40-mediated GIIS potentiation by long-chain fatty acids.

**Figure 4:**
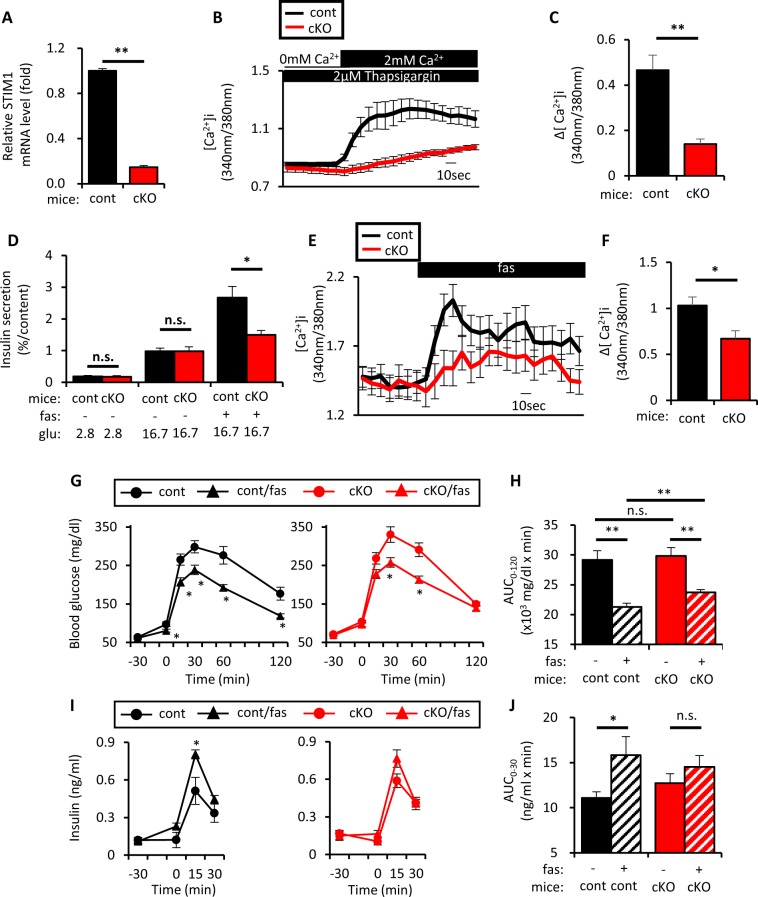
Pancreatic β-cell-specific STIM1 deficiency impaired fasiglifam-induced potentiation of insulin secretion *ex vivo* and *in vivo*. (**A**) Total RNA was extracted from islets of β-cell-specific STIM1 knockout mice (Rip-Cre+/−, STIM1fl/fl; cKO) or control littermates (Rip-Cre −/−, STIM1fl/fl; cont) and subjected to cDNA synthesis followed by real-time PCR to evaluate STIM1 mRNA expression (n = 8 per group). (**B**) Dissociated single islet cells of cKO mice or cont littermates were recorded for [Ca^2+^]_i_ to evaluate SOCE by fura-2 fluorescence ratio in response to extracellular Ca^2+^ supplementation in conditions as in (Figs 2B and 3B) (cont, n = 16; and cKO, n = 15). (**C**) Increment of fura-2 ratio (Δ[Ca^2+^]_i_) in response to extracellular Ca^2+^ supplementation in conditions as in (**B**) [(Max. value during 0–3 min) − (Average value during −1–0 min)]. (**D**) Ten isolated islets of cKO mice and cont littermates were collected in a tube and incubated at 2.8 mM or 16.7 mM glu with or without 10 μM fas for 60 min at 37 °C to evaluate insulin secretion (n = 6–7 per group). (**E**) Dissociated single islet cells of cKO mice or cont littermates were recorded for [Ca^2+^]_i_ evaluated by fura-2 fluorescence ratio in response to various stimuli (−1–0 min, 11.1 mM glu; 0–3 min; 11.1 mM glu/10 μM fas) (cont, n = 42; and cKO, n = 31). (**F**) Increment of fura-2 ratio in response to 10 μM fas in conditions as in (**E**) [(Max. value during 0–3 min) − (Average value during −1–0 min)]. (**G**,**I**) Levels of blood glucose and insulin were determined in cKO and cont littermates at 12–14 weeks of age that were fasted for 16 hours; the mice were dosed orally with 0.5% methylcellulose aqueous solution (10 mL/kg) with or without fasiglifam (fas) (30 mg/kg) 30 min before oral glucose load (2 g/kg). (cKO, n = 12; cont n = 11) cKO represented by red circle, cKO with fas represented by red triangle, cont represented by black circle, cont with fas represented by black triangle. (**H**) Area-under-the-curve of blood glucose levels in (**G**) during 0–120 min (AUC_0–120_) are shown. (**J**) Area-under-the-curve of insulin levels in (**I**) during 0–30 min (AUC_0–30_) are shown. cKO represented by red bar, cKO with fas represented by tilted lines, cont represented by black bar, cont with fas represented by *tilted* lines. Data are expressed as mean ± SEM. * and ** denote p < 0.05 and p < 0.01, respectively, by the Mann-Whitney U-test.

To address the relevance of STIM1 in fas-mediated GIIS potentiation in mice, β-cell-specific STIM1 conditional knockout (βSTIM1 cKO) mice were studied. βSTIM1 cKO mice exhibited similar bodyweight and ad lib blood glucose levels compared to littermate control mice (Fig. S3A,B). Islet morphology, β-cell mass and the α/β ratio of βSTIM1 cKO mice did not differ from those of littermate control mice (Fig. S3C–E). SOCE in islet cells isolated from βSTIM1 cKO was significantly impaired (Fig. 4A–C). Fas-mediated GIIS potentiation and [Ca^2+^]_i_ increase also were significantly impaired in islets isolated from βSTIM1 cKO mice (Fig. 4D–F), although GPR40 mRNA levels were similar in βSTIM1 cKO and control mice (Fig. S4). Notably, unlike STIM1 KD in MIN6 cells, which totally abolished fas-mediated GIIS potentiation, residual fas-enhanced GIIS occurred in islets isolated from βSTIM1 cKO mice (16.7 mM glu vs 16.7 mM glu + 10 μM fas in βSTIM1 cKO: p < 0.05) (Fig. 4D). Similar results were obtained in islets isolated from female βSTIM1 cKO mice (Fig. S5). Importantly, addition of xestospongin C to fas-mediated GIIS from islets isolated from βSTIM1 cKO mice further decreased the residual fas-mediated GIIS potentiation (Fig. S6), suggesting that the effects of STIM1 deficiency on fas-mediated GIIS potentiation might be compensated downstream of IP3Rs.

The effects of STIM1 deficiency on glucose tolerance and insulin secretion with or without fas administration were then investigated *in vivo*. In order to exclude possible effects of dietary nutrients other than glucose to more accurately assess the effect of GPR40 activation on GIIS, the mice were fasted for 16 h. Without fas administration, glucose levels and AUC-glucose during OGTT were similar in βSTIM1 cKO and control mice (Fig. 4G,H). Fas ameliorated glucose levels and AUC-glucose similarly in βSTIM1 cKO and control mice, although the effects of fas on glucose levels and AUC-glucose were significantly less in βSTIM1 cKO mice compared those in littermates (Fig. 4G,H). Without fas administration, insulin levels before and after OGTT and AUC-insulin during OGTT in βSTIM1 cKO and control mice also were similar (Fig. 4I,J). Importantly, fas significantly enhanced insulin secretion in control mice, but not in βSTIM1 cKO mice (Fig. 4I,J), indicating that the IP3R1/STIM1/Orai1 pathway plays a pivotal role in fas-mediated GIIS potentiation *in vivo*.

## Discussion

The present study reveals the critical role of the IP3R1/STIM1/Orai1 pathway in GPR40-mediated GIIS potentiation in pancreatic β-cells by showing that: (1) knockdown of IP3R1, STIM1 and Orai1 significantly impairs fas-mediated GIIS potentiation and [Ca^2+^]_i_ increase in MIN6 cells, together with significant suppression of SOCE in STIM1 and Orai1 knockdown, and (2) β-cell-specific ablation of the STIM1 gene suppresses SOCE and partially impairs fas-mediated GIIS potentiation in mice.

It is established that GPR40 activation results in the production of DAG and IP3, which promotes the release of Ca^2+^ from the ER and potentiates GIIS^13^, but it has remained unknown how Ca^2+^ release from the ER by GPR40 activation contributes to GIIS potentiation. Previous reports demonstrated in INS-1E cells and β-cells that depletion of Ca^2+^ in the ER by thapsigargin or carbachol causes STIM1 translocation^24,25^, and that dominant negative blockade of Orai1 impairs insulin secretion^26^. These findings suggest that GPR40 activates the STIM1/Orail1 pathway in pancreatic β-cells. However, there are no previous reports on the effect of STIM1 in GPR40-induced potentiation of GIIS and its effect on glucose metabolism using knockout mice. It was also previously noted that DAG activated PKD1 to promote actin depolymerization and increase insulin secretion^12^, that the GPR40 agonist fas depolarized the cell membrane and potentiated GIIS through the PLC-TRPC3 pathway^31^, and that GPR40 activation reduced the voltage-gated delayed rectifying K^+^ current, which prolonged membrane depolarization during action potential firing^32^. The relative contribution of each of these mechanisms in GPR40-mediated GIIS potentiation remains unknown. In the present study, we demonstrate a novel function of the IP3R1/STIM1/Orai1 pathway to initiate SOCE and potentiate GIIS that plays a crucial role in GPR40 signaling. Furthermore, the role of the IP3R1/STIM1/Orai1 pathway in GPR40-mediated GIIS potentiation was confirmed by use of the long-chain fatty acid palmitate.

The mechanism of GIIS potentiation by GPR40 activation remains a question. It was previously shown that IP3Rs have ATP binding sites that are activated by IP3 as well as by ATP, which is produced by glucose metabolism^33,34^. On the other hand, STIM1 and Orai1 do not have any known ATP-binding sites, and it has not been reported whether STIM1 or Orai1 activity is directly regulated by intracellular ATP. Thus, it is likely that involvement of IP3R1, rather than STIM1 or Orai1, explains why GPR40 activation enhances insulin secretion glucose-dependently. Although we demonstrated that IP3R1 plays an important role in fas-stimulated GIIS potentiation in MIN6 cells, the distribution of IP3Rs isoforms differs among cell types and species^35,36^; it remains to be determined which of the IP3R isoforms plays the pivotal role in fas-stimulated Ca^2+^ signaling in pancreatic β-cells.

The other modulator of ER Ca^2+^ storage, the SERCA pump has been well studied. It is reported that glucose promotes ATP production and causes ER Ca^2+^ filling through activation of the SERCA pump^37^. Tengholm *et al*. also reported that ATP increases the ER Ca^2+^ concentration and augments IP3-induced Ca^2+^ release^28^. This increased ER Ca^2+^ storage by the SERCA pump might also contribute to the glucose-dependent fas-induced intracellular Ca^2+^ increase. On the other hand, in the present study, inhibition of STIM1 or Orai1 abolished the effect of fas, which strongly supports the involvement of SOCE in GPR40 signaling.

cAMP is also recognized to play a role in GIIS potentiation by GLP-1 receptor activation. Yamada *et al*. demonstrated that the cAMP-PKA pathway was not involved in fas-mediated potentiation of GIIS using PKA inhibitor and measuring cAMP production^31^. Previous study found that cAMP caused STIM1 translocation; however, STIM1 did not co-localize with Orai1 and failed to activate SOCE^25^. Several studies have demonstrated that a conformational change of STIM1, which is promoted by sensing of ER Ca^2+^ storage depletion and binding of the SOAR (STIM1–Orai1 activating region) domain with Orai1, is required in addition to STIM1 translocation to activate SOCE^38–40^. This might explain the difference between the SOCE contribution to the GPR40 signal and the cAMP signal.

In this study, STIM1 KD or Orai1 KD in MIN6 cells clearly abolished fas-induced GIIS potentiation but partially impaired [Ca^2+^]_i_ increase (Figs 2E and 3E). In addition, Fas transiently increased [Ca^2+^]_i_ in the absence of extracellular Ca^2+^, suggesting fas-induced Ca^2+^ release from the ER via IP3Rs. On the other hand, fas robustly increased [Ca^2+^]_i_ in the presence of extracellular Ca^2+^, suggesting fas-induced Ca^2+^ release from the ER via IP3Rs plus SOCE via STIM1/Orai1. Thus, the partial suppression of the fas-induced [Ca^2+^]_i_ increase in STIM1 KD (Fig. 2E) or Orai1 KD cells (Fig. 3E) may be due to preserved Ca^2+^ release from the ER via IP3Rs in addition to incomplete KD of STIM1 and Orai1. The effects of STIM1 or Orai1 disruption on the ER Ca^2+^ pool have been addressed previously by studying thapsigargin-induced transient [Ca^2+^]_i_ increase, but the results are controversial. Some studies claimed that the ER Ca^2+^ pool was scarcely affected in STIM1-deficient cells or dominant negative Orai1-overexpressing cells^26,41^, while others found that ER Ca^2+^ storage was only slightly reduced in STIM1-deficient cells^21,42^. The effects of STIM1 disruption on ER Ca^2+^ storage were also investigated using the ER-targeted FRET-based probe D1ER, which showed that ER Ca^2+^ levels were slightly decreased in STIM1 KD cells^43^. Although the effects of STIM1 or Orai1 disruption on the ER Ca^2+^ pool were not addressed in the current study, it is likely that impaired SOCE, rather than potential reduction in the ER Ca^2+^ pool, underlies the impaired fas-induced [Ca^2+^]_i_ increase in STIM1 KD cells and Orai1 KD cells.

The impairment of fas-induced GIIS potentiation in isolated islets and the fas-mediated amelioration of glucose tolerance *in vivo* was mild in βSTIM1 cKO mice. It has been generally accepted that gene deletion early in life often results in various compensations. Several studies have reported that the STIM1-related protein STIM2 also mediates SOCE, and that simultaneous deletion of STIM1 and STIM2 results in a more severe phenotype in immune cells^41^. Consistent with the previous observation that STIM2 expression was upregulated in STIM1 knockout mice^23^, STIM2 mRNA in the present study was found to be slightly but significantly expressed at a higher rate in islets of βSTIM1 cKO mice (Fig. S4), which could compensate for the effect of STIM1 deletion. Furthermore, the PKC-TRPC3 pathway or the PKD pathway, which are known to be in the downstream pathway of the GPR40 signal, could also compensate for STIM1 deficiency in mice. Recently, Kono *et al*. found that STIM1 deficiency impaired insulin secretion in INS-1 832/13 cells^42^, in contrast to our current study, which found that STIM1 deficiency had little effect on GIIS by itself. However, they did not observe any compensatory increase of STIM2 in INS-1 832/13 cells lacking STIM1. Thus, it is possible that the relative abundance of STIM1 plus STIM2 may be critical in the discrepancy between our study and their study; it is of interest to investigate insulin secretion in STIM1 and STIM2 double knockout mice.

Nevertheless, fas-mediated [Ca^2+^]_i_ increase was largely impaired in the absence of extracellular Ca^2+^, and β-cell-specific STIM1 deletion severely reduced SOCE and fas-mediated GIIS potentiation in islet cells, indicating that SOCE plays an important role in GIIS potentiation by GPR40 activation.

In conclusion, the current study demonstrates that the IP3R1/STIM1/Orai1 pathway plays an important role in GPR40 agonist fas-mediated SOCE initiation and subsequent GIIS potentiation.

## Methods

### Materials

Xestospongin C, and thapsigargin were obtained from Wako (Japan). Triton-X100 and bovine serum albumin fraction V were from Nakalai Tesque (Japan). Stealth siRNA for IP3R1 (MSS275151), Silencer® Select pre-designed siRNA for STIM1 (S74488), and Orai1 (S99511) were from Thermo Fisher Scientific (USA). Fasiglifam and DAPI solution were obtained from AdoQ Bioscience (USA) and Dojindo (Japan), respectively.

### Cell culture

Mouse insulinoma cell line MIN6 cells were obtained from Dr. Jyunichi Miyazaki, and were cultured in Dulbecco’s modified Eagle’s medium (DMEM) containing 25 mM glucose (D5796; Sigma, USA) supplemented with 10% fetal bovine serum (FBS), 1 mM sodium pyruvate, 0.060 mM 2-mercaptoethanol, 100 units/ml penicillin, and 100 μg/ml streptomycin in a humidified atmosphere at 37 °C containing 5% CO_2_.

### Transfection

MIN6 cells were transfected as previously described with minor modifications^44^. Briefly, MIN6 cell suspensions were mixed with Opti-MEM™ containing siRNA and Lipofectamine® 2000, and applied to a culture dish of appropriate size to perform experiments after 48 h. For measurement of insulin secretion or preparation of total RNA, 2 × 10^5^ MIN6 cells suspended in 400 μl of DMEM without antibiotics were mixed with 100 μl of Opti-MEM™ containing 2.5 μl Lipofectamine® 2000 and 50pmol siRNA in each well of a Falcon® 24-well plate. For measurement of intracellular Ca^2+^ dynamics, 4 × 10^5^ MIN6 cells suspended in 800 μl of DMEM without antibiotics were mixed with 200 μl of Opti-MEM™ containing 5 μl Lipofectamine® 2000 and 100 pmol siRNA in a 35 mm glass bottom dish.

### Mice

Male C57BL/6 J mice were purchased from SLC Japan, Inc (Japan). Rip-Cre mice^45^ and STIM1 floxed mice^21^ were crossbred to obtain pancreatic β-cell-specific STIM1 conditional knockout (cKO) mice (i.e., STIM1^flox/flox^; Rip-Cre(+/−) mice). Male animals were housed in a 12-h light-dark cycle with free access to water and standard chow. Bodyweight (BW) and blood glucose levels were measured every week from 6 weeks of age to 13 weeks of age. All animal experimental procedures were approved by the Animal Research Committee of Kyoto University Graduate School of Medicine (MedKyo: 18249) and all experiments were performed in accordance with relevant guidelines and regulations of the Animal Research Committee of Kyoto University Graduate School of Medicine.

### Oral glucose tolerance test

Oral glucose tolerance test was performed as previously described with minor modifications^46^. Mice were dosed orally with 0.5% methylcellulose aqueous solution (10 mL/kg) with or without fas (30 mg/kg) 30 min before oral glucose load (2 g/kg). Blood samples were collected from the tail vein of mice at various time points using heparinized calibrated glass capillary tubes. Blood glucose levels were determined using Glutest Neo Sensor (Sanwa Kagaku Kenkyusho, Japan). Plasma samples prepared by centrifugation of the blood samples at 9000 g for 10 min were subjected to insulin measurement using Ultra Sensitive PLUS Mouse Insulin ELISA kit (Morinaga, Japan).

### Islet isolation

Islets of Langerhans were isolated from mice by collagenase digestion as previously described with minor modifications^47^. Briefly, Hanks’ balanced salt solution (HBSS) containing 5 mM NaHCO_3_, 20 mM HEPES, 1% BSA, and 0.5 mg/ml collagenase P (Roche, Germany) was injected to the mouse pancreas via the bile duct. The pancreas was removed and digested in a Falcon tube placed in a water bath at 37 °C for 30 min. The digested pancreas was washed twice by Krebs–Ringer bicarbonate (KRB) buffer (129.4 mM NaCl, 5.2 mM KCl, 2.7 mM CaCl_2_, 1.3 mM MgSO_4_, 24.8 mM NaHCO_3_, 1.3 mM KH_2_PO_4_) containing 2.8 mM glucose, and density gradient centrifugation was performed using histopaque 1119, histopaque 1077 and histopaque 1050 (prepared by mixing two volumes of histopaque 1077 with one volume of distilled water). After centrifugation, the islets found in the inter-phase between histopaque 1050 and histopaque 1077 were collected and transferred to a large dish. The islets were washed in ice-cold KRB buffer, hand-picked and cultured overnight in RPMI 1640 medium containing 10% FBS, 10 mM HEPES, 5 mM NaHCO_3_, 1 mM sodium pyruvate, 100 U/ml penicillin, 100 mg/ml streptomycin and 11.1 mM glucose at 37 °C in humidified air containing 5% CO_2_ before experiments.

### Quantitative reverse transcription (qRT)-polymerase chain reaction (PCR)

Total RNA was isolated from MIN6 cells or isolated islets using QIAshredder (QIAGEN) and RNeasy mini kit (QIAGEN), and subjected to cDNA synthesis using TaqMan Reverse Transcription Reagents (Thermo Fisher, USA) and quantitative real-time PCR using Power SYBR Green Master Mix (Thermo Fisher, USA) according to the manufacturers’ instructions using the StepOnePlus real-time PCR system (Applied Biosystems, USA). Gene-specific primers are listed in Table S1. Relative expression of each mRNA was calculated by the ΔΔCT method using GAPDH mRNA as invariant control as described previously^48^.

### Immunoblot analysis

MIN6 cells were transfected with siRNA as described above, and transfected MIN6 cells were then washed with phosphate-buffered saline (PBS); isolated mouse islets or homogenized lung were suspended in lysis buffer [10 mM Tris⋅HCl (pH 7.4)/100 mM NaCl/1% (wt/vol) SDS] containing protease inhibitor cocktail (Roche, Germany). Thirty micrograms of total protein were subjected to SDS-PAGE gels (Bio-Rad, USA), transferred to nitrocellulose filters, and subjected to immunoblot analysis. After blocking with PBS containing 0.1% Tween 20 and 5% skim milk at room temperature for 1 h, blotted membranes were incubated overnight at 4 °C with anti-IP3R1 antibody (8568; Cell signaling, USA) at 1:1000 dilution, anti-IP3R3 antibody (610312; BD Biosciences, USA) at 1:1000 dilution, anti-GAPDH antibody (SC-32233; Santa Cruz, USA) at 1:1000 dilution. Blotted membranes were then washed by PBS containing 0.1% Tween 20 and incubated with anti-rabbit or anti-mouse IgG secondary antibody (GE Healthcare, UK) diluted at 1:3000 at room temperature for 30 minutes before detection using ECL prime (GE Healthcare, UK). Band intensity was quantified with Multi Gauge software (Fujifilm, Japan).

### Measurement of insulin secretion

Overnight-cultured islets were pre-incubated in Buffer A [KRB buffer containing 10 mM HEPES and 0.2% BSA adjusted to pH 7.4] containing 2.8 mM glucose at 37 °C for 60 min. After preincubation, 10 size-matched islets were hand-picked up into tubes with 1 mL of Buffer A containing various stimulants and incubated at 37 °C for 60 min. After incubation, each tube was centrifuged at 9000 g for 1 min and supernatant was collected for measuring insulin secretion. After removing the remaining Buffer A, the islets were lysed in 200 μL of PBS with 0.2% Triton-X100 for measuring insulin content. siRNA-transfected MIN6 cells were then washed twice with 1 mL of Buffer A 48hrs after transfection and pre-incubated in 0.5 mL of Buffer A containing 2.8 mM glucose at 37 °C for 60 min. After preincubation, the MIN6 cells received 0.5 mL of Buffer A containing various stimulants and were incubated at 37 °C for 60 min. After incubation, supernatant was collected for measuring insulin. After removing the remaining Buffer A, the cells were lysed in 500 μL of PBS with 0.2% Triton-X100 for measuring insulin content. Insulin concentrations were determined by homogeneous time-resolved fluorescence (HTRF) assay using Insulin High Range Assay Kit (CIS Bio international, France) and Synergy H1 (BioTek Instruments, USA) according to the manufacturers’ instructions.

### Measurement of intracellular Ca^2+^ dynamics

Isolated islets were hand-picked up into a 1.5-mL tube, and dissociated into single cells in Buffer B (136.9 mM NaCl, 4.0 mM KCl, 11.9 mM NaHCO_3_, 11.1 mM glucose, 0.42 mM NaH_2_PO_4_, 0.18 mM KH_2_PO_4_). Single islet cells were seeded on a 35-mm glass bottom dish filled with RPMI1640 and cultured at 37 °C overnight before experiments. MIN6 cells were transfected with siRNA in a 35-mm glass bottom dish as described above and cultured at 37 °C for 48 hr before experiments. The single islet cells and MIN6 cells were loaded with 5 μM fura-2 AM (Dojindo, Japan) in Buffer A containing 2.8 mM glucose for 30 min at 37 °C, placed on an inverted microscope (IX71; Olympus, Japan), and observed under the 20x objective lens through the filter sets (340/26, 387/11 -DM 495–510/84). Images were obtained every 10 s; the 340 nm/380 nm fluorescence ratio in the regions of interest (ROI) were analyzed using Aqua Cosmos (Hamamatsu Photonics, Japan). The single islet cells and MIN6 cells were perifused with Buffer B containing various stimulants pre-warmed at 36.5 ± 0.5 °C using the perifusion system (WARNER Instruments, USA).

### Immunohistochemistry

Dissected pancreas of cKO and control mice were fixed by 4% paraformaldehyde and embedded in paraffin. After deparaffinization and antigen-retrieval, 3 μm-sliced samples were incubated with Buffer C [PBS with 10% goat-serum and 0.2% Trition-X100] for 30 min at room temperature, and subsequently incubated with Buffer C supplemented with rabbit anti-insulin (200-fold dilution; ab181547; Abcam, USA) and mouse anti-glucagon (2000-fold dilution; K79bB10; Abcam, USA) at room temperature overnight. The samples were then washed with PBS with 0.05% Tween 20, and incubated with Buffer C supplemented with Alexa Fluor 488-conjugated goat anti-rabbit IgG (H + L) antibody (200-fold dilution; A-11034; Thermo Fisher Scientific, USA) and Alexa Fluor 546 goat anti-mouse IgG (H + L) antibody (200-fold dilution; A-11030; Thermo Fisher Scientific, USA) for 1 hr at room temperature. The samples were washed with PBS and then incubated with PBS containing DAPI (final concentration 0.01 mg/mL) for 15 min at room temperature. The samples were then washed by PBS and mounted using Vectashield (VECTOR LABORATORIES INC., USA) on coverslips. For measuring β-cell mass, five sliced samples were prepared from each paraffin block with at least a 100μm-interval, and were observed using BZ-X700 (KEYENCE, Japan) under the x4 objective lens through the filters (470/40 -DM 495–525/50: Alexa Fluor 488) and (360/40 -DM 400–460/50: DAPI); the average ratio of insulin-positive area/DAPI-positive area was automatically calculated by BZ Analyzer (KEYENCE, Japan). β-cell mass of control and cKO mice was calculated by multiplying the insulin-positive area/DAPI-positive area ratio by pancreas weight. For measuring α-cell/β-cell ratio, images of 20 islets in each pancreas were obtained using BZ-X700 under the x20 objective lens through the filters (470/40 -DM 495–525/50: Alexa Fluor 488) and (545/25 -DM 565–605/70: Alexa Fluor 546), and the average of glucagon-positive area/insulin-positive area was manually calculated.

### Statistics

Data are expressed as mean ± standard error of the mean (SEM). Comparison between two groups was performed by the Mann-Whitney U-test. When appropriate, one-way analysis of variance (ANOVA) with post hoc analysis by the Mann-Whitney U-test was used. * And ** denote p < 0.05 and p < 0.01, respectively. The statistical analysis was carried out using SPSS Statistics 24 software (IBM Corp., Armonk, NY, USA).

## Supplementary information

Supplemental figures and table

## Data Availability

The datasets generated during the current study are available from the corresponding author upon reasonable request.
