# Supplementary material for: GPR40 activation initiates store-operated Ca2+ entry and potentiates insulin secretion via the IP3R1/STIM1/Orai1 pathway in pancreatic β-cells
Source: Sci Rep. 2019 Oct 29;9:15562. doi: 10.1038/s41598-019-52048-1 (PMC6820554; doi:10.1038/s41598-019-52048-1)

**Supplemental Materials**

GPR40 activation initiates store-operated Ca^2+^ entry and potentiates insulin secretion via the IP3R1/STIM1/Orai1 pathway in pancreatic β-cells.

Ryota Usui, Daisuke Yabe, Muhammad Fauzi, Hisanori Goto, Ainur Botagarova, Shinsuke Tokumoto, Hisato Tatsuoka, Yumiko Tahara, Shizuka Kobayashi, Toshiya Manabe, Yoshihiro Baba, Tomohiro Kurosaki, Pedro Luis Herrera, Masahito Ogura, Kazuaki Nagashima, Nobuya Inagaki

**Table S1. List of primers used in quantitative reverse transcriptase polymerase chain reaction.**

| mRNA | Forward | Reverse |
| --- | --- | --- |
| GPR40 | GGGAGCCATTCACGGGTAT | TGGACAACAGTACCAGTTC |
| IP3R1 | GGATCTAGTTCCACAAGCAGG | TGCCTCCTTCCAGAAGTG |
| IP3R2 | GAGAGAAGAGGACAGTGACG | TATGCTTTGGATGTGGCTGA |
| IP3R3 | CAGAACGACCGCAGGTTTGTCAT | TTCTGCCCATTGTTGGGAACATCG |
| GAPDH | AAATGGTGAAGGTCGGTGTG | TCGTTGATGGCAACAATCTC |
| STIM1 | TTATTGGTGTGGGTGGCTGC | ACTTCCACAGTTCGGTGCTC |
| STIM2 | ATGTCGCTGAGTCCACCTTG | TGCAGGTGACTATGTTTATTCG |
| Orai1 | TCCCTGGTCAGCCATAAGAC | TCATGGAGAAGGGCATAAGG |

**Figure S1. Protein expression levels of IP3Rs and GAPDH in mouse islets and MIN6 cells.**

(A) Western blot analysis of IP3R1 and IP3R3 expression in mouse islets and MIN6 cells. (B, C) MIN6 cells were set up for experiments on day 0 at 8x10^5^ cells per 6 well plate and transfected with 200pmol of control siRNA or IP3R1 siRNA. On day 2, the transfected MIN6 cells were lysed and immunoblotted with anti-IP3R1 Ab and anti-GAPDH Ab to determine IP3R1 expression. IP3R1 band intensity analysis for control siRNA (n=4) and IP3R1 siRNA (n = 4) adjusted by GAPDH was shown in (C). Data are expressed as mean±SEM. ** denotes p<0.01 by the Mann-Whitney U-test.

Uncropped blots were represented in Fig. S8.

**
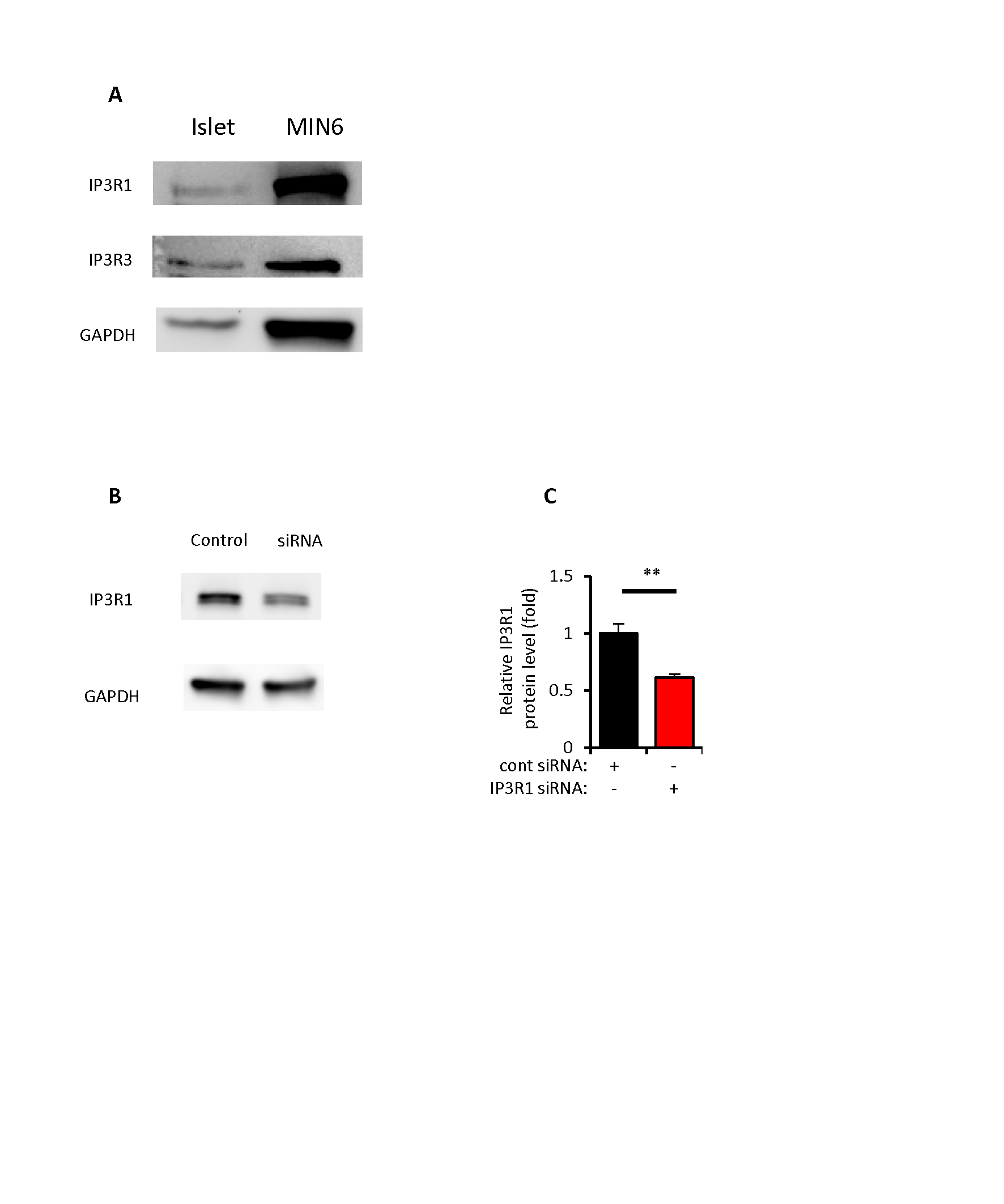
**

**Figure S2. Inhibition of IP3R1-STIM1-Orai1 pathway decreased palmitate-induced potentiation of glucose-induced insulin secretion.**

MIN6 cells were set up for experiments on day 0 at 2x10^5^ cells per 24 well plate and transfected with 50pmol of control (cont) siRNA or IP3R1 (A), STIM1(B), and Orai1(C) siRNA. On day 2, the transfected MIN6 cells were incubated in 2.8mM or 16.7mM glucose (glu) with or without 500μM palmitate for 60min at 37℃ to evaluate insulin secretion (n=5-6 per group). Data are expressed as mean±SEM. * denotes p<0.05 by the Mann-Whitney U-test.

**
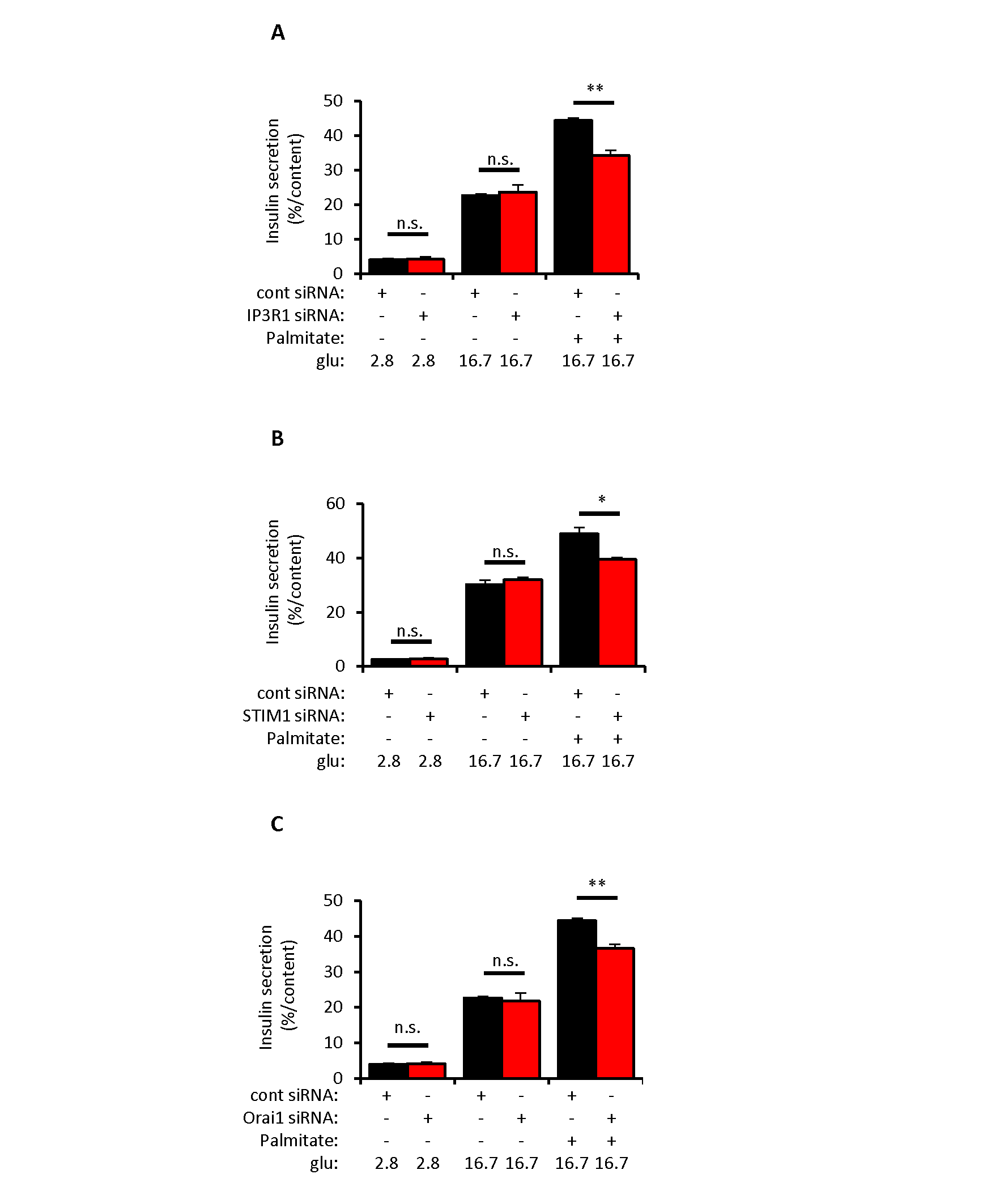
**

**Figure S3. Bodyweight, blood glucose change and immunohistochemical analysis of βSTIM1 cKO mice.**

(A) Bodyweight changes of cKO mice (n=12) and cont littermates (n=11) are shown. (B) Blood glucose levels of cKO mice (n=13) and cont littermates (n=14) are shown. (C) Sections of paraffin-embedded pancreas from cKO and cont mice at 12 weeks of age were subjected to immunostaining for insulin (green) and glucagon (red). Representative merged images are shown. (D, E) β-cell mass and α/β cell ratio of cKO and cont mice (n=4 per group) were calculated as in Methods. Data are expressed as mean±SEM.

**
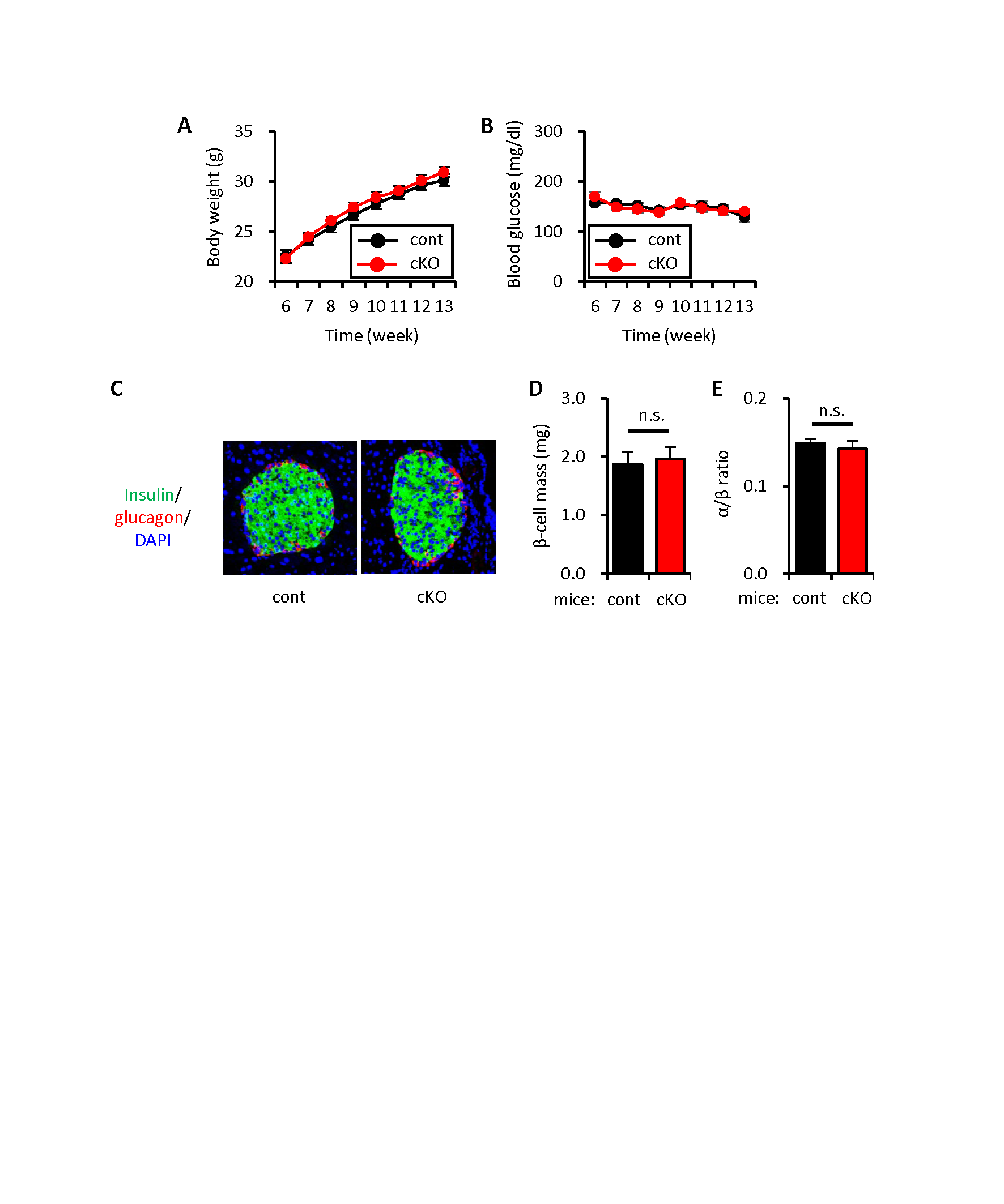
**

**Figure S4. Inositol triphosphate receptor 2 and STIM2 mRNA expression was increased in β-cell-specific STIM1 conditional knockout mice.**

Total RNA was extracted from islets of β-cell-specific STIM1 conditional knockout (cKO) mice or control (cont) littermates and subjected to cDNA synthesis followed by real-time PCR to evaluate mRNA expression of GPR40, inositol triphosphate receptor (IP3R) 1, IP3R2, IP3R3, STIM2 and Orai1 (n=8 per group). Data are expressed as mean±SEM. * denotes p<0.05 by the Mann-Whitney U-test.

**
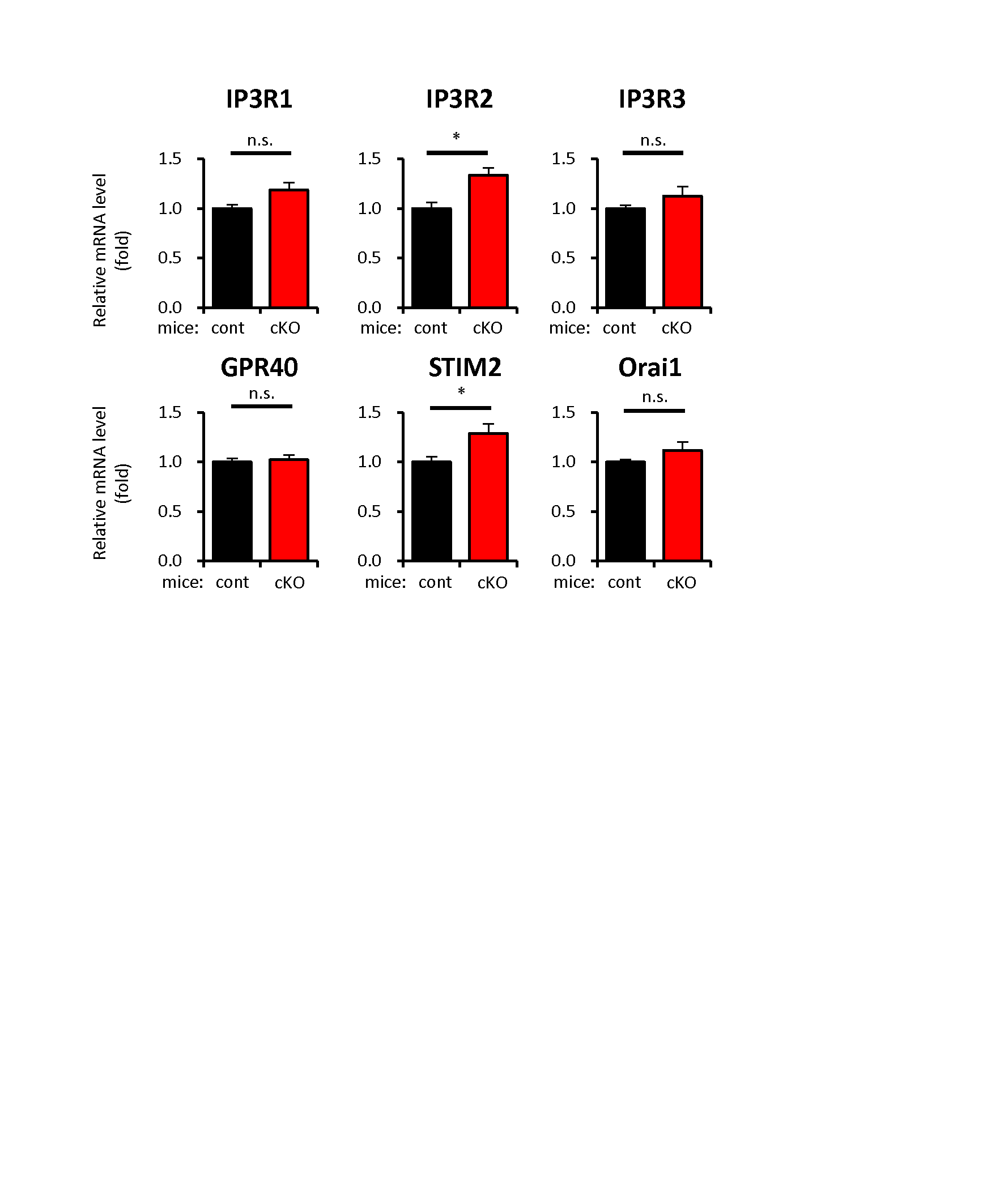
**

**Figure S5. Pancreatic islets from female β-cell-specific STIM1 conditional knockout mice also showed impaired fasiglifam-induced potentiation of insulin secretion.**

Ten isolated islets of female β-cell-specific STIM1 conditional knockout (cKO) mice and female control littermates (cont) were collected in a tube and incubated at 2.8mM or 16.7mM glucose (glu) with or without 10μM fasiglifam (fas) for 60min at 37℃ to evaluate insulin secretion (n=6-7 per group). Data are expressed as mean±SEM. * denotes p<0.05 by the Mann-Whitney U-test.

**
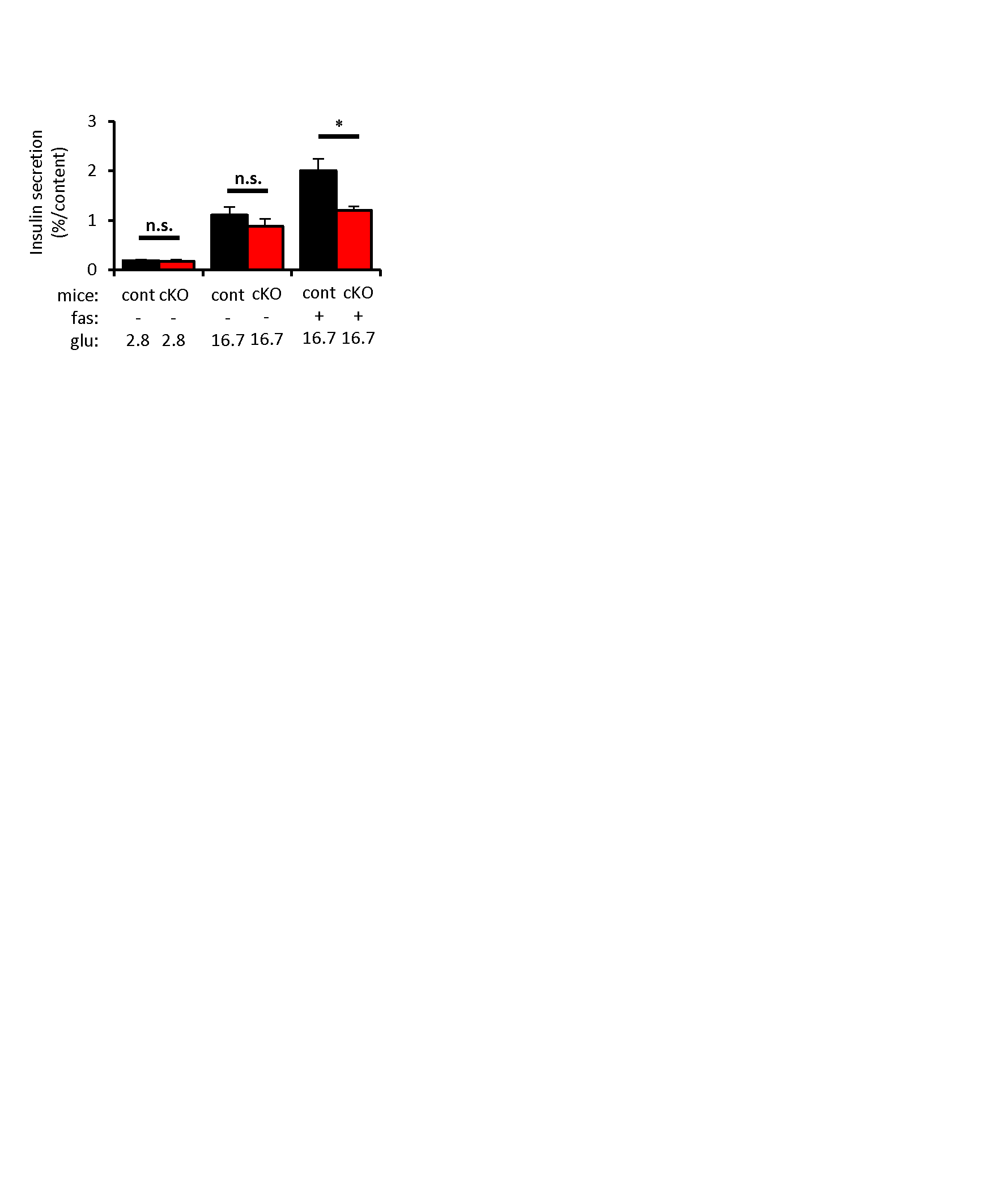
**

**Figure S6. Effect of fas and xestospongin C on insulin secretion from β-cell-specific STIM1 conditional knockout mice.**

Ten isolated islets of male β-cell-specific STIM1 conditional knockout (cKO) mice were collected in a tube and incubated at 16.7mM glucose (glu) with or without 10μM fas and 2μM xestospongin C for 60min at 37℃ to evaluate insulin secretion (n=6-7 per group). Data are expressed as mean±SEM. * denotes p<0.05, by the Mann-Whitney U-test.

**
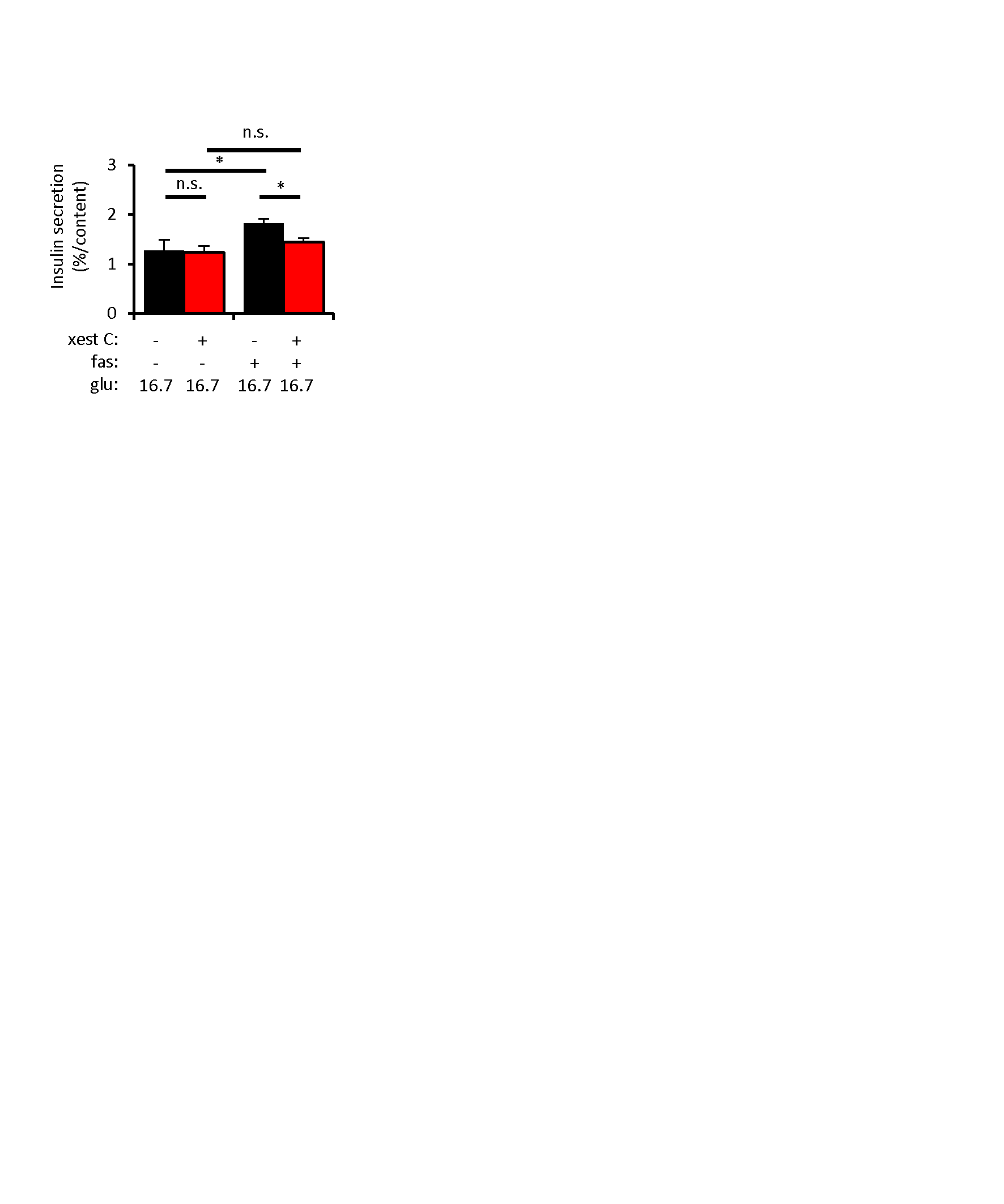
**

**Figure S7. The uncropped image of key panels in the main figure.**

Red boxes indicate the cropped portion of each image presented in the corresponding main figure.


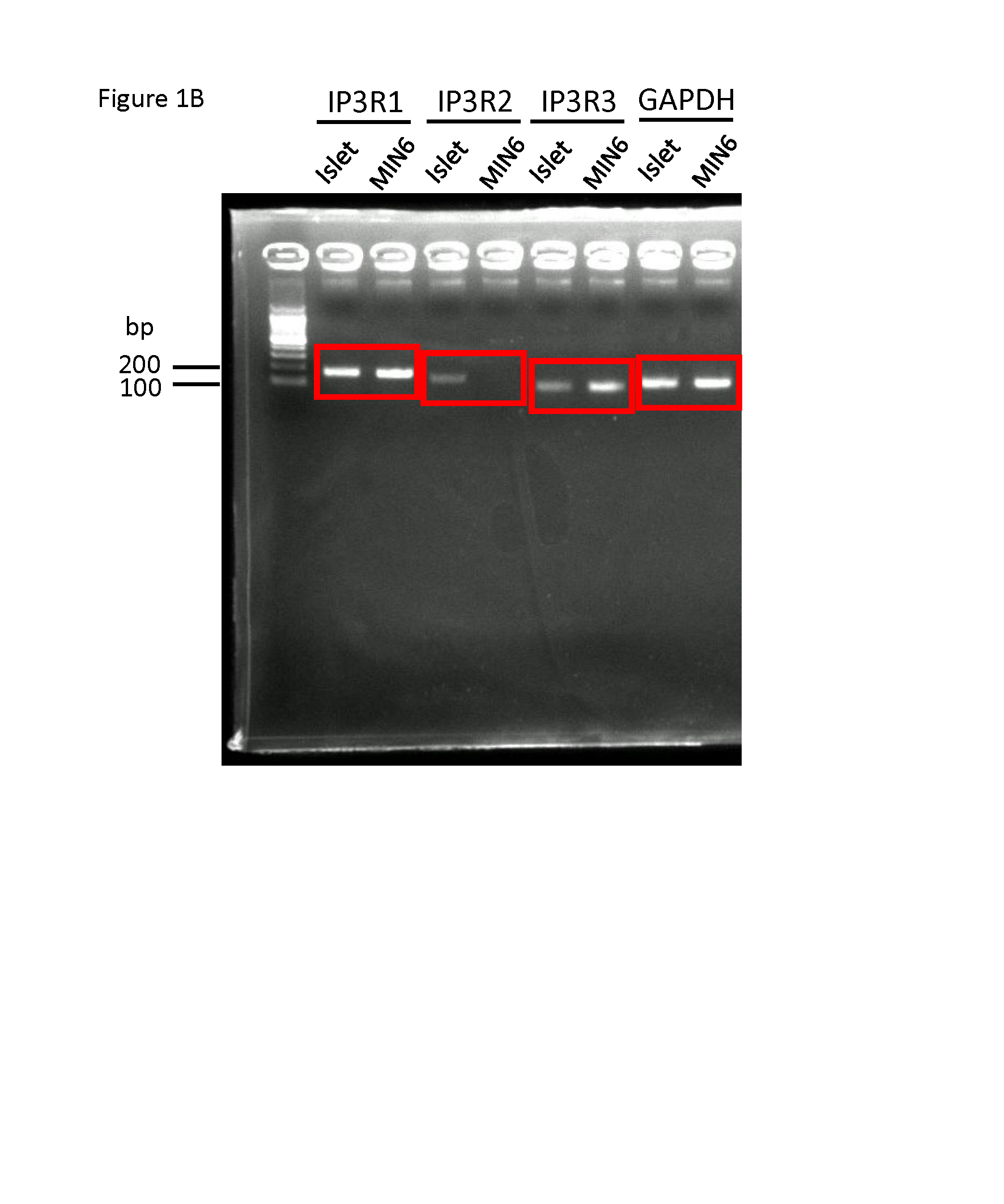


**Figure S8. The uncropped image of immunoblots.**

Red boxes indicate the cropped portion of each image presented in the corresponding immunoblot.


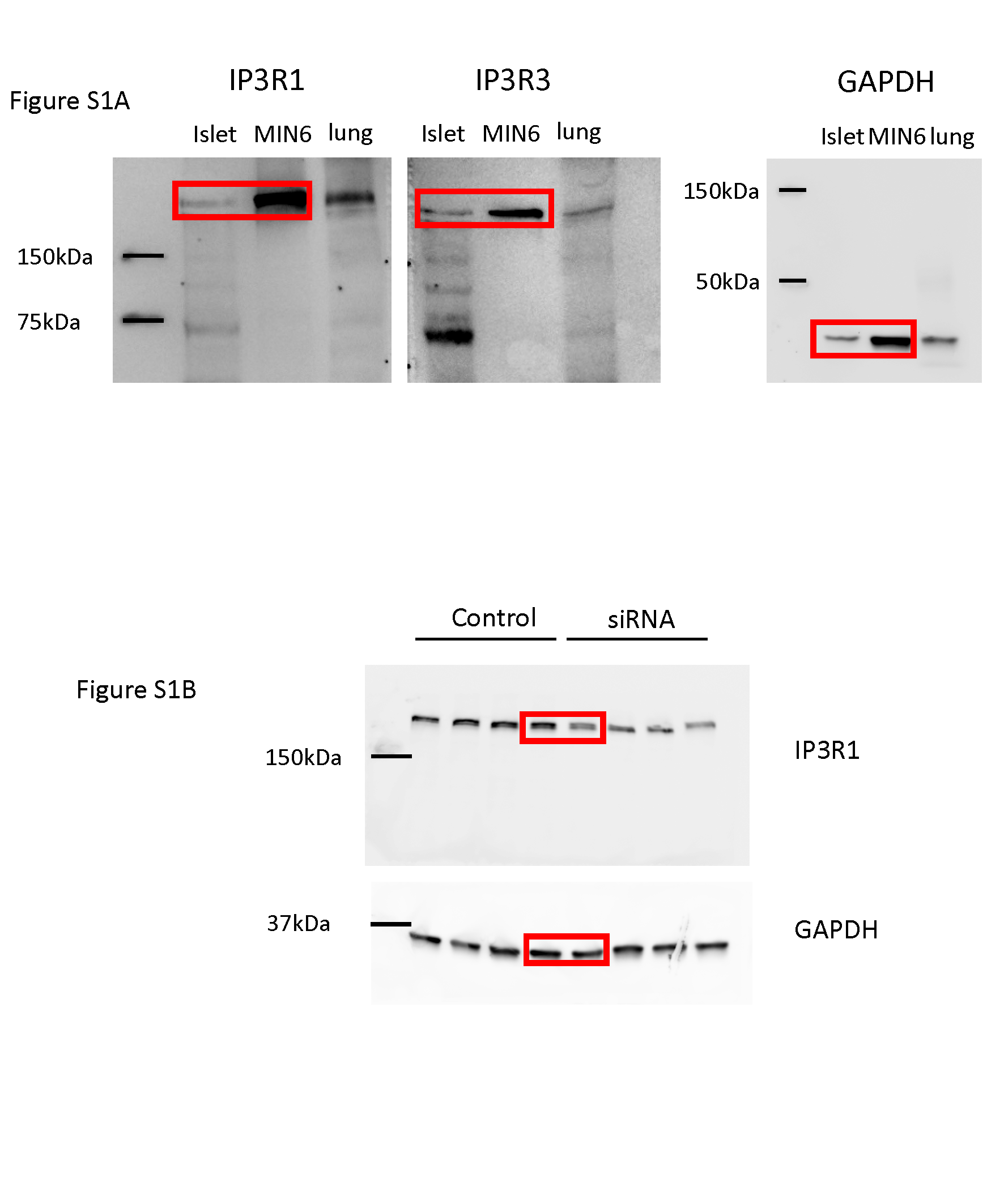

Supplement: Supplementary file 1 — Supplemental figures and table [file 41598_2019_52048_MOESM1_ESM.docx]
